# 

**Published:** 1984-10

**Authors:** 


OCTOBER 1984
VOLUME 99 (iv)
NUMBER 372

